# Nontoxigenic *Corynebacterium diphtheriae*: an emerging pathogen in England and Wales?

**DOI:** 10.3201/eid0606.000614

**Published:** 2000

**Authors:** M Reacher, M Ramsay, J White, A De Zoysa, A Efstratiou, G Mann, A Mackay, R C George

**Affiliations:** Public Health Laboratory Service, London, United Kingdom. mreacher@phls.org.uk

## Abstract

Confirmed isolates of nontoxigenic *Corynebacterium diphtheriae* in England and Wales increased substantially from 1986 to 1994. Ribotyping of 121 isolates confirmed in 1995 showed that 90 were of a single strain isolated exclusively from the throat; none had previously been identified in toxigenic strains from U.K. or non-U.K. residents. The upward trend in nontoxigenic *C. diphtheriae* probably represented increased ascertainment, although dissemination of a particular strain or clone may have been a factor.


### Nontoxigenic *Corynebacterium diphtheriae*: An Emerging Pathogen in England and Wales?

**Mark Reacher,* Mary Ramsay,* Joanne White,* Aruni De Zoysa,* Androulla Efstratiou,* Gina Mann,* Andrew Mackay,† and Robert C. George***

*Public Health Laboratory Service, London, United Kingdom; †Greenwich District General Hospital, Vanbrugh Hill, London, United Kingdom

Confirmed isolates of nontoxigenic *Corynebacterium diphtheriae* in England and Wales increased substantially from 1986 to 1994. Ribotyping of 121 isolates confirmed in 1995 showed that 90 were of a single strain isolated exclusively from the throat; none had previously been identified in toxigenic strains from U.K. or non-U.K. residents. The upward trend in nontoxigenic *C. diphtheriae* probably represented increased ascertainment, although dissemination of a particular strain or clone may have been a factor.

Clusters of cases of sore throat associated with isolation of nontoxigenic *Corynbacterium diphtheriae* were detected in gay men attending a genitourinary medicine clinic, military recruits, and children from a religious community in England and Wales in the late 1980s to mid-1990s (1-4). To determine the public health importance of the increase in cases, the Public Health Laboratory Service's (PHLS's) Streptococcus and Diphtheria Reference Unit and the PHLS Communicable Disease Surveillance Centre obtained more complete clinical information on nontoxigenic isolates referred in 1995 and 1996. Isolates received in 1995 were further characterized by molecular typing (ribotyping).

### The Study

Laboratories in England and Wales routinely submit isolates of *C. diphtheriae* to the PHLS Streptococcus and Diphtheria Reference Unit for confirmation and toxin determination by both phenotypic (Elek and other immunoassays) and genotypic (polymerase chain reaction) methods (5,6). Routine screening of throat swabs with selective culture media for *C. diphtheriae* was encouraged in public health laboratories in England and Wales (7). Clinical and epidemiologic information was obtained from questionnaires sent to referring laboratories and from laboratory request forms. Responsibility for completion and return of the enhanced surveillance questionnaires was taken by laboratory staff, in consultation with senior medical microbiologists and attending physicians with access to laboratory and medical records. The questionnaires, which included history of recent travel, symptoms and signs of illness, general medical history, clinical management (particularly antibiotic treatment, contact tracing, and treatment of contacts) and bacteriologic and virologic investigations, were sent retrospectively for isolates received by the PHLS diphtheria unit from January to June 1995 and prospectively through 1996 (8). Isolates confirmed as nontoxigenic *C. diphtheriae* during 1995 were ribotyped. The isolates were referred from laboratories in England, Wales, Scotland, the Channel Islands, and the Isle of Man. Analysis of ribotype patterns was done by using Taxotron (Institut Pasteur, France) software, as previously described (9).

In 1995 and 1996, PHLS confirmed 265 isolates from residents of England and Wales as nontoxigenic *C. diphtheriae* (Figure 1). The isolates were submitted by 80 laboratories located throughout each country; 28 (35%) were public health laboratories. Each laboratory submitted 1 to 27 isolates. Questionnaires were

Address for correspondence: M. Reacher, Public Health Laboratory Service, Communicable Disease Surveillance Centre, 61 Colindale Avenue, London NW9 5EQ, U.K.; fax: 44-0-20-8200-7868; e-mail: mreacher@phls.org.uk.

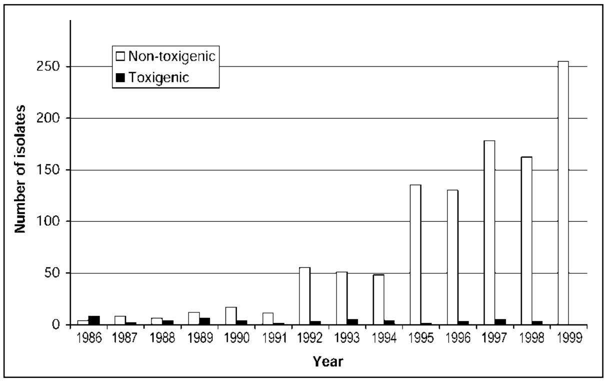

Figure 1. Annual number of isolates of *Corynebacterium diphtheriae* confirmed by the Public Health Laboratory Service's Streptococcus and Diphtheria Reference Unit, from residents of England and Wales, 1986-1999.

returned for 236 (89%) isolates. No pharyngeal membrane or systemic toxicity was reported. The age range of patients whose isolates were tested was 1 to 87 (median 20) years.

Two hundred forty-seven (93%) isolates were from the throat, ten (4%) from skin lesions, one from blood, one from a nose swab, and one from bronchial washings; five were from unrecorded sites. Biotype var g*ravis* accounted for 223 (84%), var *mitis* for 33(12%), and var *belfanti* for 9 (3%) isolates.

Of the 247 throat isolates, 238 were obtained as a result of clinical evaluation of patients with sore throats, and 9 were obtained from contacts of these patients. Of the 238 isolates obtained during evaluations, more than 25% were from male attendees at general outpatient or genitourinary medicine outpatient clinics (Table 1). Of isolates from females patients, 7% were from general outpatient clinics, and none were from genitourinary medicine outpatient clinics. Most isolates were from 15- to 24-year-old patients (Table 2). Of 238 throat isolates, 29 (12%) were from patients who had traveled outside the United Kingdom in the preceding 3 months, 20 to Western Europe, 4 to Australasia, 2 to Africa, 2 to the Indian subcontinent, and 1 to the Caribbean. Fever, lymphadenopathy, or both were reported in association with 72 (30%) of throat isolates. Nontoxigenic *C. diphtheriae* was reported as the predominant organism in 171 (72%) of the 238 throat swabs (Table 3) but was mixed with beta-hemolytic streptococci in 67 (28%). Penicillin was prescribed for 100 patients and a macrolide for 66, for a total of 166 (70%) patients treated according to current U.K. guidelines (10). Other antibiotics were prescribed for 7 patients and none for the rest. Viral throat cultures, reported for 10 (4%) of the 238 patients, were negative.

Table 1. English and Welsh patients with sore throats whose throat swabs yielded nontoxigenic *Corynebacterium diphtheriae,* by sex, 1995 and 1996

| | Male patients | | Female patients | | Not recorded | | Total | |
|---|---|---|---|---|---|---|---|---|
| Clinical setting | No. | % | No. | % | No. | % | No. | % |
| General practice | 50 | 52 | 98 | 74 | 3 | 38 | 151 | 63 |
| Outpatients | 15 | 15 | 9 | 7 | 1 | 13 | 25 | 11 |
| GUM clinic[a] | 11 | 11 | 0 | 0 | 1 | 13 | 12 | 5 |
| Inpatients | 8 | 8 | 6 | 5 | 0 | 0 | 14 | 6 |
| Other | 2 | 2 | 5 | 4 | 0 | 0 | 7 | 3 |
| Not recorded | 11 | 11 | 15 | 11 | 3 | 38 | 29 | 12 |
| All settings | 97 | 100 | 133 | 100 | 8 | 100 | 238 | 100 |

[a]GUM = genitourinary medicine.

Table 2. English and Welsh patients with sore throats whose throat swabs yielded nontoxigenic *Corynebacterium diphtheriae*, by age, 1995 and 1996

| | Age | | | | | | | | | |
|---|---|---|---|---|---|---|---|---|---|---|
| | <15 | | 15-24 | | 25-34 | | 35+ | | All ages | |
| Clinical Setting | No. | % | No. | % | No. | % | No. | % | No. | % |
| General practice | 20 | 13 | 103 | 68 | 24 | 16 | 4 | 3 | 151 | 100 |
| Outpatients | 1 | 4 | 15 | 58 | 7 | 31 | 2 | 8 | 25 | 100 |
| GUM clinic[a] | 0 | 0 | 6 | 50 | 4 | 33 | 2 | 17 | 12 | 100 |
| Inpatients | 4 | 29 | 8 | 57 | 2 | 14 | 0 | 0 | 14 | 100 |
| Other | 0 | 0 | 2 | 29 | 4 | 57 | 1 | 14 | 7 | 100 |
| Not recorded | 5 | 17 | 14 | 47 | 8 | 30 | 2 | 7 | 29 | 100 |
| All settings | 30 | 13 | 148 | 62 | 49 | 21 | 11 | 5 | 238 | 100 |

[a]GUM = genitourinary medicine.

Table 3. Pathogens in mixed growth with nontoxigenic *Corynebacterium diphtheriae* in throat swabs from English and Welsh patients with sore throats, 1995 and 1996

| Pathogen | No. | % |
|---|---|---|
| Lancefield Group A streptococci | 26 | 11 |
| Lancefield Group C streptococci | 30 | 13 |
| Lancefield Group G streptococci | 11 | 5 |
| None | 171 | 72 |
| Total | 238 | 100 |

Positive serologic results for infectious mononucleosis were reported in 10 (4%) patients. Seven isolates were associated with HIV infection, eight with psoriasis, one with gonorrhea, two with malaria, and one with cytomegalovirus infection and Crohn disease. Four of five laboratories referring 10 or more isolates reported that they had screened all throat swabs with selective media for *C. diphtheriae* during 1995 and 1996. These laboratories were located at two teaching hospitals in central London (25 and 19 isolates) and two public health laboratories, one in the northwest of England (13 isolates) and the other in Wales (10 isolates). These 67 isolates were obtained from screening 32,345 throat swabs during the survey period. This rate corresponds to an overall rate of two isolates per thousand throat swabs (1.2 to 2.9 isolates per thousand throat swabs for each individual laboratory). Of the 10 skin isolates, 2 were var *gravis*, 7 var *mitis*, and 1 var *belfanti*. Nine were associated with travel outside the United Kingdom in the previous 3 months: to Africa (three patients), the Indian subcontinent (one patient), the Caribbean (two patients), and Southeast Asia (three patients). The positive blood culture was biotype var *mitis*, obtained from a 2-year-old with congenital heart disease whose illness was diagnosed as endocarditis 3 weeks after returning from Pakistan. The isolate from the nose was var *belfanti* mixed with *Klebsiella aerogenes*, taken from a 23-year-old man of Pakistani origin, who had a 3-month history of rhinitis but had not traveled in the preceding 3 months. The isolate from bronchial washings was var *belfanti*, associated with a malignant lung tumor in a 68-year-old man.

Taxotron analysis was undertaken in 121 (90%) of 135 specimens obtained for isolation from referrals from laboratories in all eight health regions in England and Wales during 1995; 115 of these had been obtained through clinical evaluation and 6 through contact tracing (Table 4). Travel outside the United Kingdom in the preceding 3 months was reported in association with isolates from 17 patients (Tables 4 and 5). Eight additional isolates that had been submitted by laboratories in Scotland, the Channel Islands, and the Isle of Man were ribotyped (Table 4). Twenty-three distinct patterns were detected and were designated A to W (Figure 2 and Tables 4 and 5). Ribotypes A, B, C, and D were biotype *gravis*; ribotypes E, F, G,

Table 4. Throat isolates of nontoxigenic *Corynebacterium diphtheriae* submitted to the Public Health Laboratory Service's Streptococcus and Diphtheria Reference Unit, from U.K. residents,[a] 1995

| Site of isolate | Travel outside U.K. within previous 3 months | No. ribotyped | Ribotypes |
|---|---|---|---|
| Isolates from residents of England and Wales | | | |
| Throat | No | 96 | 78A,3B,1C,1D,5F,1G,1I,1J,1L,1N,1O,1P,1T |
| Throat | Yes | 11 | 7A,1B,1E,1F,1U |
| Skin | Yes | 5 | 1D,1H,1J,1M,1W |
| Blood | Yes | 1 | 1Q |
| Bronchial washings | No | 1 | 1S |
| Nose | No | 1 | 1V |
| Total 115 | | | |
| Isolates from contacts of residents of England and Wales | | | |
| Throat | No | 6 | 5A,1Q |
| Total | | 121 | |
| Isolates from residents of Scotland, Channel Islands, and Isle of Man | | | |
| Throat | No | 8 | 4A,1K,1L,2R |
| Total U.K. | | 129 | |

[a]U.K. = England, Wales, Scotland, Channel Islands, and Isle of Man.

Table 5. Nontoxigenic *Corynebacterium diphtheriae* isolates from patients who traveled outside England and Wales in the previous 3 months, submitted to the Public Health Research Laboratory's Streptococcus and Diphtheria Reference Unit, 1995

| Residence[a] | Destination | Ribotype | Site |
|---|---|---|---|
| London | Australia | A | Throat |
| London | Sierra Leone | A | Throat |
| Northwest | Cyprus | A | Throat |
| Northwest | Holland | A | Throat |
| Northwest | Canary Islands | A | Throat |
| Northwest | Spain | A | Throat |
| Wales | Germany | A | Throat |
| London | France | B | Throat |
| London | Vietnam | D | Skin |
| London | Gambia | E | Throat |
| London | Canary Islands | F | Throat |
| London | Sudan | H | Skin |
| Southeast | Philippines | J | Skin |
| London | Ghana | M | Skin |
| West Midlands | Pakistan | Q | Blood |
| London | Morocco | U | Throat |
| West Midlands | Jamaica | W | Skin |

[a]English Health Regions and Wales.

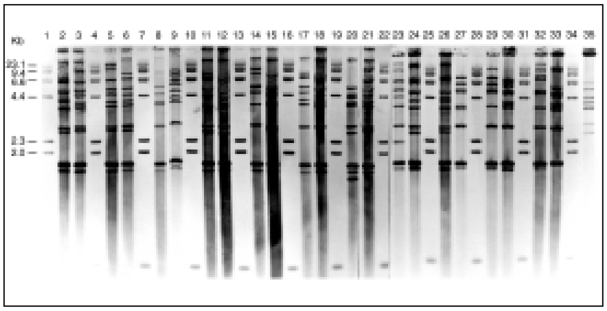

Figure 2. *Bst*EII rRNA gene profiles of nontoxigenic *Corynebacterium diphtheriae* from isolates submitted to the Public Health Laboratory Service's Streptococcus and Diphtheria Reference Unit from U.K. residents,* 1995. Lanes 1, 4, 7, 10, 13, 16, 19, 22, 25, 28, 31, and 34 contain lambda *Hin*dIII digests as size standard (sizes indicated on left). The remaining tracks show ribotypes A to W: lane 2, 95/13 (A); lane 3, 95/281 (B); lane 5, 95/384 (C); lane 6, 95/358 (D); lane 8, 95/220 (E); lane 9, 95/258 (F); lane 11, 95/277 (G); lane 12, 95/515 (H); lane 14, 95/173 (I); lane 15, 95/171 (J); lane 17, 95/382 (K); lane 18, 95/167 (L); lane 20, 95/340 (M); lane 21, 95/428 (N); lane 23, 95/424 (O); lane 24, 95/453 (P); lane 26, 95/418 (Q); lane 27, 95/23 (R); lane 29, 95/338 (S); lane 30, 95/18 (T); lane 32, 95/324 (U); lane 33, 95/389 (V); lane 35, 95/21 (W).
U.K. = England, Wales, Scotland, Channel Islands, and Isle of Man.

H, I, J, K, L, M, N, O, P, and Q were biotype *mitis*; and ribotypes R, S, T, U, V, and W were biotype *belfanti*.

Ribotype A was isolated only from the throat and accounted for 90 (74%) of 121 isolates from residents of England and Wales (Table 4). The isolates represented 78 of the 96 throat isolates from specimens obtained during clinical evaluation and not associated with recent travel, 7 of 11 throat isolates taken at investigation and associated with recent travel, and 5 of 6 throat isolates obtained at contact tracing (Tables 4 and 5). Ribotype A also accounted for 4 of 8 throat isolates obtained at investigation from residents of Scotland, the Channel Islands, and Isle of Man (Table 4).

The remaining ribotype strains accounted for one to five isolates and were associated with a single health region or with travel outside the United Kingdom (Tables 4 and 5). Ribotype E was isolated from the throat of one person who had recently traveled to Gambia (Table 5). The five ribotypes from skin isolates were related to travel (Table 5); ribotypes H, M, and W were present in single isolates, while ribotypes D and J were also detected in nontravel-related throat isolates. Ribotype Q was isolated from blood of the 2-year-old with congenital heart disease and from the throat of a 4-year-old sibling. The nose isolate from the 23-year-old patient of Pakistani origin was ribotype V, and that obtained from bronchial washings of the 68-year-old man was ribotype S. All ribotypes from contacts were the same as their index case isolates. Ribotypes A, G, N, Q, P, L, and H formed a cluster with a genetic distance of <0.2, indicating greater than 80% genetic homology. Ribotypes L and H exhibited more than 90% genetic similarity.

### Conclusions

One invasive infection with *C. diphtheriae* was associated with known risk factors. A single case of endocarditis without recognized risk factors was reported in England immediately before the survey (11). This picture is similar to that of an Australian series in which three of seven cases of invasive infection had no predisposing risk factors (12). Most isolates were from throat swabs of young adults in primary care. The preponderance of female patients reflects the pattern of age- and sex-specific consultation rates for acute pharyngitis and tonsillitis (ICD 9th Revision codes 462 and 463) seen in general practice (13). Men attending genitourinary medicine clinics accounted for 5% of throat isolates, but there were no isolates in female patients from such settings. This is consistent with clustering previously noted in gay men but could be due to laboratory practice in hospitals serving large gay populations.

It is not known whether nontoxigenic *C. diphtheriae* strains were responsible for the illnesses that prompted the study. More than 25% of the 238 throat isolates obtained at investigation of sore throats were associated with beta-hemolytic streptococcal infection, infectious mononucleosis, or another illness; negative results for viral culture of the throat and for infectious mononucleosis were reported in a small proportion of the remainder. Community-based carriage studies and case-control studies, supported by comprehensive virologic investigation, will be required to obtain more complete information on the pathogenicity or copathogenicity of nontoxigenic *C. diphtheriae* in throat isolates.

Current U.K. guidelines state that when identified, nontoxigenic *C. diphtheriae* be regarded as a potential pathogen and be treated with penicillin or erythromycin if the patient has symptoms (10). Treatment was generally in accordance with these guidelines, but contact tracing, undertaken in 68 (26%) patients, and administration of a diphtheria immunization booster to 18 (8%) patients were not recommended. The few nontoxigenic *C. diphtheriae* isolates associated with chronic skin ulcers were mainly associated with recent travel to tropical zones.

A total of 23 distinct ribotypes were observed. However, ribotype A accounted for most isolates, was isolated exclusively from the throat, and was detected in isolates obtained throughout the United Kingdom. Seven of eleven throat isolates associated with a recent history of travel were also ribotype A, and it is possible that these were acquired in the U.K. Ribotype A predominated and appeared to circulate freely within the U.K. in 1995, which suggests that this strain may have some advantage in terms of transmissibility or pathogenic potential.

If nontoxigenic strains of *C. diptheriae* vary in factors associated with increased transmissibility of pathogenic potential, toxigenic strains may also vary in these factors. Toxigenic strains with these factors could be more likely than toxigenic strains without these factors to produce epidemics. This type of relationship may explain the appearance of an epidemic clone in the Russian diphtheria epidemic of the 1990s.

The marked variation in number of nontoxigenic *C. diphtheriae* isolates referred by laboratories in different regions probably reflected differences in the use of selective culture media for *C. diphtheriae* and practice in referral of isolates to PHLS. Increased professional awareness of the risk for imported diphtheria during the 1990s would have been expected to have increased both of these factors and may explain most, if not all, of the increase in the number of nontoxigenic *C. diphtheriae* isolates ascertained by the PHLS Streptococcus and Diphtheria Reference Unit during this period (Figure 1).

It has been suggested that nontoxigenic strains could become toxigenic by acquiring the *tox* gene, assuming that the chromosomal diphtheria toxin repressor gene (*dtx*R) is functional (14-16). However, no reports of membrane or systemic toxicity were received for any of our isolates, and ribotype patterns in the U.K. isolates for toxigenic and nontoxigenic strains differed. The rise in nontoxigenic strains from 1985 to 1996 and thereafter has not been accompanied by a rise in toxigenic isolates (Figure 1). These observations suggest that conversion to toxin production had not occurred despite continuing circulation of nontoxigenic strains. However, documented introductions of toxigenic *C. diphtheriae* into the U.K. are extremely rare.

Results from the four laboratories that routinely screen all throat isolates with selective culture media indicated a low isolation rate. This may not be seen as a cost-effective activity by many laboratories; less biased and cost-effective surveillance data could be obtained by undertaking selective culture for *C. diphtheriae* in population-based samples, accompanied by strict compliance with reporting.

Our data confirm the known association of nontoxigenic strains with localized disease and with occasional cases of invasive infection, particularly endocarditis. There was no evidence that nontoxigenic *C. diphtheriae* would have posed an increasing threat to public health in England and Wales during the survey period.

### Acknowledgments

We are grateful to colleagues in England and Wales for referring isolates, completing enhanced surveillance questionnaires, and providing information on laboratory workload; and to the staff of the PHLS Streptococcus and Diphtheria Reference Unit.

Dr. Reacher is consultant medical epidemiologist at the Public Health Laboratory Service Communicable Disease Surveillance Centre in London. His current responsibilities are national surveillance for bacteremia and associated antibiotic resistance, gastrointestinal infections, and training.

### References

1. Wilson APR, Efstratiou A, Weaver E, Allason-Jones E, Bingham J, Ridgway GL, et al. Unusual non-toxigenic *Corynebacterium diphtheriae* in homosexual men. Lancet 1992;339:998.
2. Efstratiou A, George R, Begg NT. Non-toxigenic *Corynebacterium diphtheriae* var *gravis* in England. Lancet 1993;341:1592-3.
3. Wilson APR. The return of *Corynebacterium diphtheriae*: the rise of non-toxigenic strains. J Hosp Infect 1995;30 Suppl: 306-12.
4. Efstratiou A, George RC. Microbiology and epidemiology of diphtheria. Reviews in Medical Microbiology 1996;7:31-42.
5. Efstratiou A, Maple PAC. Manual for the laboratory diagnosis of diphtheria. Copenhagen: World Health Organisation; 1994:ICP/EPI 038 (C).
6. Efstratiou A, George RC. Laboratory guidelines for the diagnosis of infections caused by *Corynebacterium diphtheriae* and *C. ulcerans*. Commun Dis Public Health 1999;2:250-7.
7. Public Health Laboratory Service. PHLS clinical microbiological standard operating procedure for the investigation of throat swabs. London: Technical Services, Public Health Laboratory Service Headquarters; 1996. SOP 9: issue 2.
8. Enhanced surveillance of non-toxigenic *Corynebacterium diphtheriae* infections. Comm Dis Rep CDR Wkly 1996;6:29.
9. De Zoysa A, Efstratiou A, George RC, Jahkola M, Vuopio-Varkila J, Deshevoi S, et al. Molecular epidemiology of *Corynebacterium diphtheriae* from northwestern Russia and surrounding countries studied by using ribotyping and pulsed field gel electrophoresis. J Clin Microbiol 1995;33:1080-3.
10. Bonnet JM, Begg NT. Control of diphtheria: guidance for consultants in communicable disease control. Commun Dis Public Health 1999;2:242-9.
11. Booth LV, Ellis C, Wale MC, Vyas S, Lowes JA. An atypical case of *Corynebacterium diphtheriae* endocarditis and subsequent outbreak control measures. J Infect 1995;31:63-5.
12. Tiley SM, Kokiuba KR, Heron LG, Munro R. Infective endocarditis due to nontoxigenic *Corynebacterium diphtheriae*: report of seven cases and review. Clin Infect Dis 1993;16:271-5.
13. McCormick A, Fleming D, Charlton J. Morbidity statistics from General Practice: Fourth national survey 1991-1992. London: Office of Population, Census and Surveys. Her Majesty's Stationery Office; 1995: p. 250.
14. Pappenheimer AM, Murphy JR. Studies on the molecular epidemiology of diphtheria. Lancet 1983;ii:923-6.
15. Groman NB, Cianciotto N, Bjorn M, Rabin M. Detection and expression of DNA homologous to the tox gene in nontoxigenic isolates of *Corynebacterium diphtheriae*. Infect Immun 1983;42:48-56.
16. Cianciotto NP, Groman NB. Characterization of bacteriophages from tox-containing, non-toxigenic isolates of *Corynebacterium diphtheriae*. Microb Pathog 1997;22:343-51.
